# Regular Music Exposure in Juvenile Rats Facilitates Conditioned Fear Extinction and Reduces Anxiety after Foot Shock in Adulthood

**DOI:** 10.1155/2019/8740674

**Published:** 2019-07-14

**Authors:** Si Chen, Tuo Liang, Fiona H. Zhou, Ye Cao, Chao Wang, Fei-Yifan Wang, Fang Li, Xin-Fu Zhou, Jian-Yi Zhang, Chang-Qi Li

**Affiliations:** ^1^Department of Neurology, Xiangya Hospital, Central South University, Xiangya Road 88, Changsha, Hunan 410008, China; ^2^Department of Anatomy and Neurobiology, Xiangya School of Medicine, Central South University, Tongzipo Road 172, Changsha, Hunan 410013, China; ^3^School of Biological Science and Technology, Central South University, Tongzipo Road 172, Changsha, Hunan 410013, China; ^4^Sansom Institute, School of Pharmacology and Medical Sciences, University of South Australia, Adelaide 5001, Australia; ^5^Xiang-Ya College of Medicine, Central South University, Tongzipo Road 172, Changsha, Hunan, China

## Abstract

Music exposure is known to play a positive role in learning and memory and can be a complementary treatment for anxiety and fear. However, whether juvenile music exposure affects adult behavior is not known. Two-week-old Sprague-Dawley rats were exposed to music for 2 hours daily or to background noise (controls) for a period of 3 weeks. At 60 days of age, rats were subjected to auditory fear conditioning, fear extinction training, and anxiety-like behavior assessments or to anterior cingulate cortex (ACC) brain-derived neurotrophic factor (BDNF) assays. We found that the music-exposed rats showed significantly less freezing behaviors during fear extinction training and spent more time in the open arm of the elevated plus maze after fear conditioning when compared with the control rats. Moreover, the BDNF levels in the ACC in the music group were significantly higher than those of the controls with the fear conditioning session. This result suggests that music exposure in juvenile rats decreases anxiety-like behaviors, facilitates fear extinction, and increases BDNF levels in the ACC in adulthood after a stressful event.

## 1. Introduction

It has been believed that music is able to exhibit a nonnegligible effect upon human emotion, cognition, and learning [1–4]. This concept has already been demonstrated with a series of experiments showing the behavioral, physiological, and psychological effects of human musical engagement [5–8]. Accumulating evidence in human studies has confirmed the partial efficacy of music therapy in many diseases, including schizophrenia [9], multiple sclerosis [10], and anxiety and pain [11].

In addition to the beneficial role of music in brain development, the positive effects of music on anxiety and fear extinction are thought to be able to affect cognitive ability and result in increased mental and physical comfort [12, 13]. In humans, music has been found to decrease the stress response and improve recovery from critical illness or surgery. Music imagery programs reduce anxiety in acute leukemia patients and lead to lower initial distress [5]; music therapy also has a positive effect on mood in poststroke patients [7], reduces pain response [14], and helps manage chronic pain, anxiety, and depression [6, 8, 11]. In mice, music exposure for 30 days increases levels of fear-motivated memory, resulting in changes in hundreds of genes [15]. Auditory enrichment by means of classical music is suggested to be a reliable method for reducing stress levels in several breeds of egg-laying chicks [16]. On a molecular level, music has showed increased dopamine release in the striatal system in humans [17] and significant increased dopamine synthesis in young chicks [18] and the rats [19–21], while dopamine has been shown to be one of the most important neuromodulators of fear and anxiety [22]. These studies suggest that anxiety, fear, and other stress-related disorders, as well as their underlying neurobiological mechanisms, may be partially alleviated with exposure to music. However, the mechanisms underlying the beneficial effects of music exposure are not fully understood.

On the other hand, the juvenile period is a critical developmental stage in which the environment plays an important role in future stress coping in adult rats [23, 24]. Stressful experiences in the juvenile period render animals less resistant and resilient to stressors in adulthood [25–28]. However, it is not known whether an enriched environment—including exposure to music in the juvenile stage—affords better coping in adulthood.

Basic auditory functions, in humans, such as pitch discrimination and complex melody recognition, exist in fetuses and infants [29], and appropriate vibroacoustic stimulation by exposure to music alters fetal behavior and is carried forward into the newborn period [30, 31]. Maternal exposure to music during pregnancy influences neonatal behavior in humans [32]. In animals, prenatal music exposure has been shown to improve postnatal spatial learning and memory and to reduce isolation stress [33] in chicks. Music exposure in the perinatal period reduces the error rate of adult rats in a plus maze test, increases cortical expression of TrkB and PDK1 (which are negatively correlated with test error rate) [34], and improves fear learning [35] and emotional behavior [36]. These findings indicate that music exposure during early development modulates the effect of stress on anxiety and fear extinction in adulthood.

Then, the molecular basis underlying the possible behavioral changes in adulthood associated with juvenile music exposure is supposed to be revealed. In neurosciences fields, a number of previous studies have shown that brain-derived neurotrophic factor (BDNF) plays an important role in music-induced plasticity and is a key mediator between early experiences and the adult phenotype [13, 37–40]. Meanwhile, previous studies have found that the anterior cingulate cortex (ACC) is necessary for the long-term memory and plays a significant role in the formation and consolidation of contextual fear memory as well as the fear extinction, both in mice [41] and in rats [42, 43], indicating a prominent role of the ACC in stabilizing a new memory.

Thus, the first aim of the present study was to assess the effect of music exposure in juvenile rats on anxiety-like behaviors and conditioned fear extinction following electrical stimulation of the paws in adulthood (ambient noise is treated as control) [15]. Besides, some findings indicate that music listening or auditory stimulation influences human psychobiological stress levels [44] and processes communication signal [45] in a sex-dependent manner. Sex was another source of variation in our research. Additionally, as music is a noninvasive, nonpharmacological, and culturally acceptable intervention, the effects of music on animal behavior and/or physiology may be a matter of the physical features embedded in the particular music (tempo, intensity, regularity, rhythm, or melody), and Mozart's sonata for two pianos (K. 488) was already used by other studies to observe neurophysiological and behavioral changes in scientific research [13, 20, 21, 46, 47], also in our study. The second aim was to investigate the molecular basis of the behavioral changes associated with music exposure, especially the expression of BDNF, which is the substrate regulating mood and affection, in the ACC in response to music exposure and fear conditioning.

## 2. Materials and Methods

### 2.1. Subjects

The experimental protocol was approved by the Animal Care and Use Committee of Central South University according to the National Institutes of Health Guide for the Care and Use of Laboratory Animals. Seventy-eight juvenile rats (42 male and 36 female, 180-220 g weight) were randomly divided into music group (received music exposure) and control group (ambient noise exposure only, without exposure to music), selected from litters of six SD (Sprague Dawley) mother rats obtained from Central South University Animal Services (ethics approval number 201310125, Changsha, China). Animals were raised under standard conditions of a 12 h light/dark cycle (illumination time 7 am to 7 pm), 20±2°C, 50-55% humidity, and they were allowed free access to water and food. After postnatal day (PND) 35, male and female rats were separated, and groups of four littermates were housed together throughout the experiment.

### 2.2. Experimental Design

**Figure 1:**
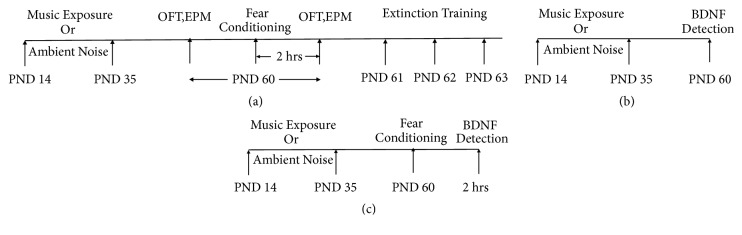
Experimental procedure. Rats were randomly assigned to the music groups or the control groups (ambient noise only). (a) Anxiety-like behaviors tests. (b, c) BDNF detection.

**Figure 2:**
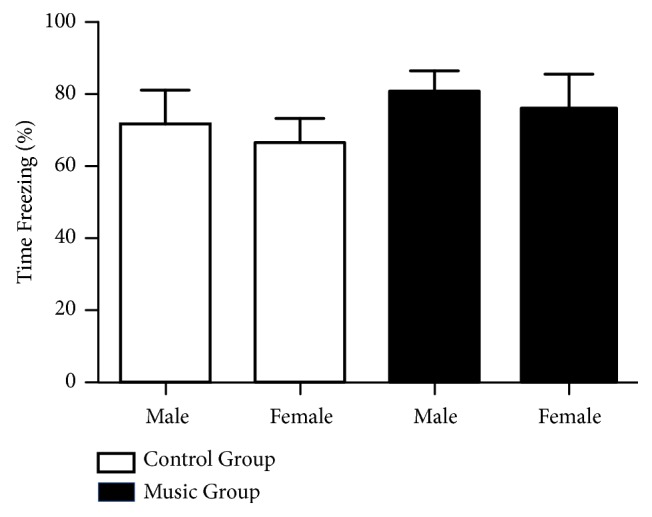
The percentage of time spent freezing (Time freezing %) in the first block of the first fear extinction training day (means ± SEM) between the music and control groups (for each group, *n*_*male*_ = 9, *n*_*female*_ = 6).

The experimental procedure is outlined in Figure 1. All animal behavior tests were conducted under blinded conditions, and the treatment groups were only revealed after all experimental data were obtained. There were two serials in the experimental design.

(A) Anxiety-like behaviors tests (Figure 1(a)): thirty juvenile rats (18 males and 12 females) were randomly divided into music group and control group. Each group consisted of 9 male and 6 female juvenile rats. After music exposure (or ambient noise exposure) from PND 14 to 35, anxiety-like behaviors in all rats were examined using the elevated plus maze test (EPM) and the open field test (OFT) prior to and 2 h after fear conditioning with footshock on PND 60. After that, the rats were submitted to fear extinction training for 3 consecutive days starting on PND 61 (PND 61, 62, 63).

(B) BDNF detection (Figures 1(b) and 1(c)): forty-eight juvenile rats (24 males and 24 females) were used in BDNF detection without anxiety-like behaviors tests. Rats were randomly divided into four groups (music with/without fear, control with/without fear). Each group consisted of 6 male and 6 female juvenile rats. After music exposure (or ambient noise exposure) from PND 14 to 35, the protein and mRNA levels of BDNF in the ACC were evaluated without (Figure 1(b)) or 2 h after fear conditioning with footshock (Figure 1(c)) on PND 60, respectively, using ELISA with BDNF-specific antibodies and real-time RT-PCR with BDNF-specific primers.

### 2.3. Music Exposure Test

On PND 14, both male and female pups were randomly assigned to one of two groups. The first music group was exposed to Mozart's piano sonata, K.488 with a sound intensity of 65-75 dB; the second (control) group was placed in a similar room without music (ambient noise; ~55 dB). Pups from each group were transported to a soundproof room and exposed to either music or ambient noise from 8 pm to 10 pm during the dark cycle for 21 consecutive days. At the end of each music/ambient noise exposure, the animals were returned to their home cage.

### 2.4. Fear Conditioning

Situated in a sound-attenuated cabinet with a red ambient light (15 W), the fear conditioning chamber consisted of a ligneous chamber (500×500×300 mm) and a grid floor. Acoustic conditioned stimuli (CS) were delivered through a speaker attached on the cabinet ceiling, whereas electrical foot shocks, which served as the unconditioned stimuli (US), were delivered through the grid floor connected to a computer-controlled shocker unit. The chamber was thoroughly cleaned with 70% aqueous ethanol between each experiment. Before each fear conditioning experiment, the rats were familiarized in the conditioning chamber for 5 min. A neutral tone (10 s; 60 dB in sound pressure level; 4 kH_Z_) along with an electrical foot shock (1 s; 1 mA) was presented 20 times at an average interval of 1 min. The rats were returned to their home cage 1 min after the final shock was applied.

### 2.5. Fear Extinction Training

After fear conditioning experiments on PND 60, the rats were submitted to fear extinction training for 3 consecutive days starting on PND 61. Each day, the rats were moved to the same conditioning chamber. After habituation for 5 min, ten tones of the CS were presented without electric foot shock. The freezing response time (defined as time in somatomotor immobility not including breathing movements) after each CS was recorded. Extinction training consisted of 5 tests wherein 2 CS were presented to the rats with an 80-s resting period between test intervals. The mean value of the freezing response time from 5 trials of fear extinction training was used for statistical analyses.

### 2.6. Anxiety-Like Behavior Tests

Anxiety-like behaviors were examined using the EPM and OFT, as described previously [48]. Briefly, in the EPM test, the rats were placed in the middle of an apparatus comprising two open arms and two closed arms that extended from a common central platform. Behavior was recorded for 10 min with a video camera. The percentage of time spent in the open arms and the number of open arm entries were recorded. In the OFT, animals were placed into one corner of an open field, which was divided into a grid of 8 × 8 squares. Movements of the animal in the area were recorded for 10-min. Exploration was defined as the time spent in the inner squares, and overall activity was defined as the number of squares crossed during the testing session.

### 2.7. BDNF Detection

The animals were euthanized by overdosing with ether and with rapid cervical dislocation. The whole brain of each rat was collected and rinsed with cold 1X PBS. Then, ACC was isolated on a cold plate. The tissue samples were frozen in liquid nitrogen and stored at −80°C.

### 2.8. BDNF Enzyme-Linked Immunosorbent Assay (ELISA)

ACC tissues were homogenized in a cold lysis buffer (137 mM NaCl, 20 mM Tris–HCl buffer, pH 8.0, 1% Nonidet-P40, 10% glycerol, and 1 mM phenyl methyl sulfonyl fluoride (PMSF)). The homogenates were centrifuged, and the supernatants were collected and stored at −20°C prior to BDNF ELISA analysis. Amounts of BDNF from each ACC sample were determined using ELISA according to the manufacturer's instructions on 96-well plates (R&D systems China, Shanghai, PRC). The optical density of the assay was measured at 450 nm using a microplate reader (Rayto RT-6000, China). BDNF concentrations were determined using the regression line obtained from the BDNF standard incubated under similar conditions in each assay. The values were expressed as pg/mg wet weight, and all assays were performed in triplicate.

### 2.9. RNA Isolation and Quantitative Real-Time PCR

Total RNA from the ACC was extracted using Trizol reagent (Invitrogen), following the manufacturer's recommendation. The RNA samples were dissolved in 30 *μ*l of DEPC-H_2_O and subsequently stored at −80°C. The quantification and purity of the RNA were measured using a NanoDrop 2000 Spectrophotometer (Thermo-Fisher Scientific, CA, USA). The quality of RNA was assessed by absorbance at 260 and 280 nm, and a ratio of A260 / A280 ranging from 1.9 to 2.1 was considered acceptable. Total RNA was transcribed using a high capacity cDNA reverse transcription kit (K1622, Fermentas, EU) according to the manufacturer's protocol. Real-time PCR was performed in a MiniOpticon Two-Color Real-Time PCR Detection System (Bio-Rad, USA) using SsoFastEvaGreensupermix (Bio-Rad). The primer sequences used in the real-time PCR reactions were as follows: BDNF Forward Primer: 5′-AAAACCATAAGGACGCGGACTT-3′, Reverse Primer: 5′-AAAGAGCAGAGGAGG CTCCAA-3′; and GAPDH Forward Primer: 5′-ACCACCATGGAGAAGGCTGG-3′, and Reverse Primer: 5′-CTCAGTGTAGCCCAGGATGC-3′. Primer specificity was confirmed by melting curve analysis and by electrophoresis of PCR products on a 2% agarose gel to confirm the presence of a single band of the predicted size. The relative expression of target gene mRNA was normalized to the amount of housekeeping gene GAPDH in same cDNA of animals from the different treatment groups and was compared to the control group without shock using the relative quantification method (2^—△△^CT method) as described by the manufacturer (Bio-Rad).

### 2.10. Statistical Analysis

(i) Kolmogorov-Smirnov test was used to evaluate the data distribution (normality); (ii) F-max tests were used to evaluate the homoscedasticity of the data. The sample size of music and control groups is the same. The homoscedasticity was confirmed to be equal. Significant differences were determined using one-way or three-way ANOVA with repeated measures, followed by Tukey's post hoc test for multiple comparisons. For OFT/EPM in a three-way ANOVA, factors are music, sex, and fear conditioning. For freezing time, factors are music, sex, and training day. For BDNF detection, factors are music, sex, and fear conditioning. Unpaired two-tailed Student's t test was used if there were only two groups. P < 0.05 was considered statistically significant.

## 3. Results

To characterize the potential roles of music exposure in juvenile rats on stress responses in adulthood, we examined auditory fear conditioning, fear extinction training, and anxiety-like behaviors (assessed with the EPM and OFT), and we investigated alterations of BDNF protein and mRNA levels in the ACC associated with music exposure.

### 3.1. Freezing Behavior in the First Block of Fear Extinction Training Day 1

As shown in Figure 2, the percentage of freezing during the first block of the first fear extinction training day was not significantly different between the music groups (male: 80.60±8.87%; female: 75.81±11.98%) and the control groups (male: 71.76±13.84%; female: 66.61±7.95%) (*F *(3, 26) = 0.537,* p = *0.6611, male* n *= 9, female* n* = 6). There was no effect of music treatment (*F *(1, 26) = 1.204,* p =* 0.2826), sex (*F *(1, 26) = 0.3662,* p* = 0.5503), or their interaction (*F *(1, 26) = 0.000,* p =* 0.9828) on the freezing time. The results suggest that music exposure in juvenile rats does not affect conditioned fear memory in adulthood.

### 3.2. Freezing Behaviors across the 3 Days of Fear Extinction Training

**Figure 3:**
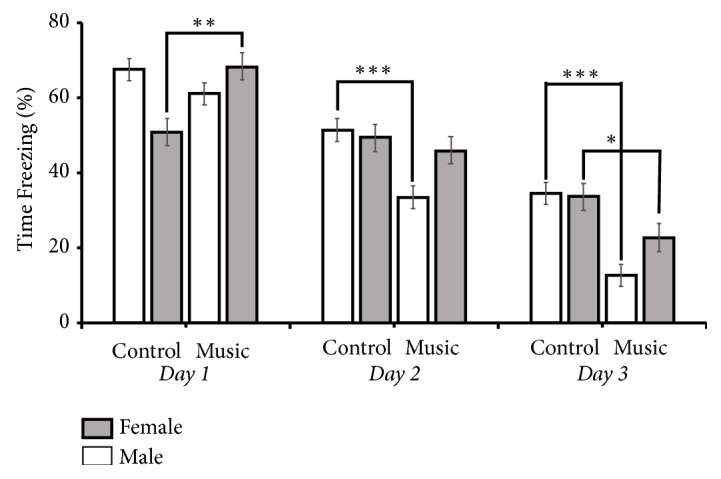
The percentage of time spent freezing (Time freezing %) across the 3 days of fear extinction training (means ± SEM) between the music and control groups (for each group, *n*_*male*_ = 9, *n*_*female*_ = 6; *∗p*< 0.05, *∗∗p*< 0.01).

As shown in Figure 3 and Table 1 (supporting information), there was a significant decrease in freezing behaviors across the 3 days of fear extinction training (*F *(2,24) = 48.60,* p*<0.0001 in male music group;* F *(2,24) = 52.71,* p*<0.0001 in male control group;* F *(2,15) = 30.61,* p*<0.0001 in female music group; and* F *(2,15) = 9.071,* p =* 0.0026 in female control group). The rats in the music group showed significantly less freezing during fear extinction training than did the rats in the control group (*F *(1,78) = 13.849,* p*< 0.001). A three-way ANOVA indicated that there was a significant effect of training day (*F *(2, 78) = 118.249,* p*< 0.001 and music treatment (*F *(1,78) = 13.849,* p*< 0.001) on freezing time. There were also significant interactions between training day and music treatment (*F *(2,78) = 11.910,* p*< 0.001) as well as between sex and music treatment (*F *(1,78) = 18.546,* p*< 0.001). There was no main effect of sex (*F *(1,78) = 0.766,* p *= 0.384) on freezing time. And there were no interactions of training day, sex, and music treatment (*F *(2,78) = 1.007,* p *= 0.370) on freezing time. These data indicate that music exposure in the juvenile rats facilitated conditioned fear extinction in adulthood.

### 3.3. Anxiety-Like Behaviors after Foot Shocks Stress

**Figure 4:**
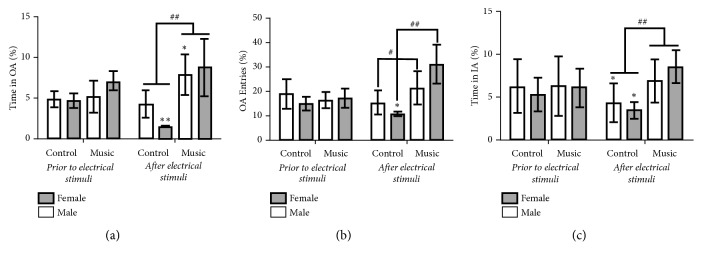
Frequency indexes of anxiety-like behavior testing following foot shock stress. (a) The time spent in the open arms (Time in OA %) in the EPM test. (b) The number of open arm entries (OA Entries %) in the EPM test. (c) The time spent in the inner area (Time in IA %) in the OFT. (OA= open arms, IA = inner area; values expressed in (means ± SEM)%; for each group, *n*_*male*_ = 9, *n*_*female*_ = 6; *∗p*< 0.05, *∗∗p*< 0.01 versus corresponding group of without shock; #*p*< 0.05, ##*p*< 0.01 music groups versus corresponding control groups after shock).

The results of the tests of anxiety-like behaviors are shown in Figure 4 and the Bonferroni-adjusted significance tests (corrected p value) and uncorrected p value by the number of comparisons made are indicated in Table 2 of supporting information. Without fear conditioning, there were no significant differences in the time spent in the open arms between the music group (male: 5.08±0.61%; female: 7.26±1.51%) and the control group (male: 4.94±0.31%; female: 4.66±0.42%) (*F *(3, 26) = 2.156,* p =* 0.117). No differences were found in the open arm entries (male: 15.66±3.44%, female: 17.91±4.34% in music group; male: 18.51±5.65%, female: 15.13±2.64% in control group) (*F *(3, 26) = 1.23,* p =* 0.358) in the EPM test, nor in the time spent in the inner area (male: 6.26±3.47%, female: 5.69±2.58% in music group; male: 6.22±3.13%, female: 5.39±1.93% in control group) (*F *(3, 26) =0.144,* p =* 0.932) in the OFT between the two groups. After foot shocks, the time spent in the open arms (male: 7.79±2.34%, female: 8.78±3.35% in music group; male: 4.29±1.57%, female: 1.51±0.12% in control group) and the number of open arm entries (male: 21.33±6.31%, female: 29.44±8.87% in music group; male: 15.42±4.76%, female: 10.44±1.04% in control group) in the music group were significantly longer than those in the control group (time spent in open arms* F *(3, 26) = 15.886,* p*<0.001, open arm entries (*F *(3, 26) = 12.376,* p*<0.001). Three-way ANOVA indicated that there was a significant effect of music treatment on the time spent in the open arms (*F *(1, 52) = 49.086,* p*<0.001), and on the number of open arm entries (*F *(1, 52) = 21.262,* p*<0.001). The OFT revealed decreased time spent in the inner area in the control rats (*F *(3, 26) = 4.162,* p =* 0.013). The rats in the music group (male: 6.95±2.46%; female: 8.03±2.25%) spent a significantly longer time in the inner area than did the controls (male: 4.36±2.22%; female: 3.40±1.10%) (*F *(3, 26) = 6.808,* p = *0.002). A three-way ANOVA indicated that there was a significant effect of music treatment on the time spent in the inner area (*F *(1,52) = 8.640,* p = *0.005) and a significant interaction between music treatment and foot shock (*F *(1, 52) = 6.397,* p =* 0.015). These results suggest that anxiety levels in the music group were significantly lower than those in the control group in both males and females after fear conditioning.

### 3.4. BDNF Protein and mRNA Levels in the ACC

**Figure 5:**
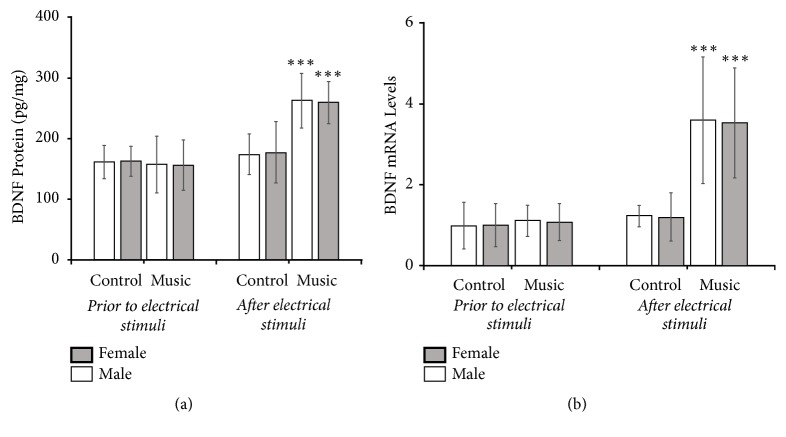
BDNF protein (a) and mRNA levels (b) in the ACC. *∗∗∗p*< 0.001 music groups versus corresponding control groups with shock (for each group, *n*_*male*_ = 6, n_female_ = 6).

The results of the BDNF protein and mRNA level analyses in the ACC are shown in Figure 5 and the Bonferroni-adjusted significance tests (corrected p value) and uncorrected p value by the number of comparisons made are indicated in Table 3 of the supporting information. Without fear conditioning, there were no differences in BDNF protein (male: 157.34±19.08 pg/mg, female: 156.05±16.60 pg/mg in music group; male: 160.87±11.26 pg/mg; female: 162.22±9.94 pg/mg in control group) (*F *(3, 20) = 0.039,* p =* 0.989,* n *= 6) or BDNF mRNA (male: 1.11±0.15, female: 1.09±0.45 in music group; male: 1.00±0.35; female: 1.00±0.52 in control group) (*F *(3, 20) = 0.055,* p =* 0.982,* n *= 6) between the music group and the control group. However, 2 h after fear conditioning, BDNF levels in the ACC in the music group (male: 262.29±18.27 pg/mg, female: 259.65±14.09 pg/mg in BDNF protein; male: 3.60±2.04, female: 3.53±1.34 in BDNF mRNA) were higher than those in the control group (male: 173.88±19.08 pg/mg, female: 177.14±20.41 pg/mg in BDNF protein; male: 1.22±0.11, female: 1.20±0.59 in BDNF mRNA) (BDNF protein:* F *(3, 20) = 8.601,* p =* 0.001,* n *= 6; BDNF mRNA:* F *(3, 20) = 5.057,* p =* 0.009,* n *= 6). A three-way ANOVA with BDNF protein level as the dependent variable indicated that there were significant effects of music treatment (*F *(1, 40) = 13.004,* p = *0.001) and fear conditioning (*F *(1, 40) = 27.974,* p*<0.001) and a significant interaction between music treatment and fear conditioning (*F *(1, 40) = 16.318,* p*<0.001). No sex effects or other interactions between variables were found. A three-way ANOVA with BDNF mRNA levels as the dependent variable indicated that there were significant effects of music treatment (*F *(1, 40) = 14.183,* p = *0.001) and fear conditioning (*F *(1, 40) = 16.862,* p*<0.001) and a significant interaction between music treatment and fear conditioning (*F *(1, 40) = 12.053,* p =* 0.001). No sex effects or other interactions between variables were found. These results suggest that BDNF levels in the ACC of the music group were significantly increased after the fear conditioning session, but no significant changes in BDNF levels in the ACC of the control group were observed following the fear conditioning session.

## 4. Discussion

In our study, we investigated the influence of music exposure in juvenile rats on fear extinction and anxiety-like behaviors induced by foot shock stress in adulthood. Our results demonstrate that music exposure in the juvenile age range could promote conditioned fear extinction and decrease anxiety-like behaviors induced by foot shock in adulthood. In particular, BDNF levels in the ACC in the music group were significantly increased after the fear conditioning session and were higher than those of the controls.

Many studies have demonstrated that enriched environments in early development and in the adulthood can ameliorate a number of behavioral symptoms, including sensory hypersensitivity, anxiety, impairments in social behavior, and alterations in the hypothalamic pituitary adrenal axis, all of which are associated with neurodevelopmental disorders [49–51]. An enriched environment is defined as a combination of complex inanimate and social stimulation. It is believed that the interaction of multiple environmental factors, rather than any single element, contributes to better cognitive functioning [52]. In comparison to white noise, music was a significant contributor to an “enriched” environment. In early development, music exposure in the perinatal period influences fetal and neonatal behaviors in humans [30–32], improves postnatal spatial learning and memory, and reduces isolation stress [33] and EPM error rates [34] in animals. The present study showed that exposing juvenile rats to music led to significant decreases in anxiety-like behaviors in adulthood after foot shock; furthermore, this music exposure promoted conditioned fear extinction. These results suggest that an enriched environment involving music exposure in the juvenile stage positively affects emotional responses to foot shock stress in adulthood.

However, there are contradictory hypotheses in the literature regarding whether long-term exposure to music can positively regulate the response of subjects to stressful adverse events. While most studies support a positive role for music exposure in the regulation of the stress response in later life, other researchers believe that the regulatory role of music is immediate and that long-term exposure to music may enhance susceptibility to traumatic stress and difficulty in coping with adverse events in rats [53]. A previous study has shown that a period of music exposure did not affect the anxiety-like behaviors of mice [15]. Here we show that, without fear conditioning, there were no differences in anxiety-like behaviors between music-exposed and control rats. This initial finding indicates that music exposure has no effect on basal levels of anxiety-like behaviors. However, after conditioned fear training, the anxiety-like level in the music group was significantly lower than that in the control as revealed by increased open-arm dwelling and entries in the EPM test and increased central area time in the OFT, indicating that juvenile music exposure contributes to adult emotional self-regulation and stability after trauma. Furthermore, we found that juvenile music exposure significantly decreased the percentage of freezing in the following days when compared with the control group, indicating that music exposure facilitates learned fear extinction after the initial foot shock. Whether this learned fear extinction contributes to the decrease in anxiety-like behaviors was not tested in our study, but our study suggests that juvenile music exposure may promote learning and memory and reduce the affective response to stressful events in adulthood. The mechanism of juvenile music exposure on the long-term behaviors of adult subjects may rely on neuronal plasticity in the brain induced by music. Several studies have shown that music exposure may mainly contribute to neurogenesis and synaptic plasticity [54] through enhanced BDNF expression in the cerebral cortex, hippocampus, hypothalamus, brain stem, and other regions [34, 55]. Moreover, music exposure affects emotional function by alterations in neurotransmitters and receptors, such as GABAA receptors, 5-HT [56], and dopamine [19]. Recently, accumulating evidence has suggested that epigenetic mechanisms, which modify chromatin structure and exert ultimate control over gene expression, may represent the molecular mechanism underlying the long-term effects of early environment on brain function and behavior [57].

BDNF plays a key role in neuronal plasticity related to life experience, learning, and memory. Previous studies have suggested that the environment during the first weeks of life permanently affects BDNF expression in rats through epigenetic mechanisms. For instance, BDNF levels in adulthood are decreased following longer exposure to stress [58], posttraumatic stress disorder (PTSD)-like behavioral stress [59], or adverse early experiences [60, 61]. In contrast, its expression is increased after learning [62], mild exercise [63], or exposure to mild stress [64]. Indeed, BDNF, which is produced and released by neurons in an activity-dependent manner, has been implicated in synaptic plasticity, learning, and memory formation. BDNF plays a role in the amelioration of learned fear disorders [65] and the extinction of conditioned fear [66] and is thus important for emotional learning. Here, we show that BDNF levels in the music group in the ACC were significantly increased after a fear conditioning session, further suggesting that exposure to music such as Mozart's piano sonata, K. 488, in the juvenile age is associated with a notable increase in BDNF expression in response to a later stressful event. Notably, there were no differences in BDNF mRNA and protein in the ACC between the music group and the control group without the foot shock. Our data indicate that music exposure in the juvenile age prepares the adult brain for future stress by encouraging the upregulation of the BDNF gene. Our data also suggest that upregulated BDNF may participate in the promotion of conditioned fear extinction and the reduction of anxiety-like behaviors. It is worth noting that previous studies have found that the ACC plays a key role in the formation and consolidation of contextual fear memory, both in mice [41, 43] and in rats [42, 43], indicating a prominent role for the ACC in stabilizing a new memory. Our finding that BDNF levels increase faster in the ACC after learned conditioned fear extinction in the music treated rats is consistent with these studies, suggesting the ACC and its expression of BDNF are important in fear learning and memory.

## 5. Conclusion

We show that continuous exposure to music during the juvenile period not only enhances conditioned fear extinction and reduces anxiety-like behaviors but also increases BDNF levels in the ACC in adult rats after stressful foot shocks. Our study expands our knowledge about how and why the juvenile stage and the juvenile environment are very important for future mental health in adulthood. Our findings further suggest that an enriched environment, including music exposure, during the juvenile stage is critical for future emotional health and can be a potential strategy for prevention of negative outcomes after stressors in adulthood.

## Data Availability

All profiling data are available as Supplementary Materials, Tables S1–S3. Other experimental data will be available upon request.

## Supplementary Materials

Supplementary Materials Table-S1: the percentage of time spent freezing across the 3 days of extinction training. Table-S2: the Bonferroni-adjusted significance tests (corrected p value) and uncorrected p value by the number of comparisons made in the anxiety-like behavior testing were shown as the time spent in the open arms (A), the number of open arm entries (B) in the EPM test, and the time spent in the inner area in the open field test (C). Table-S3: the Bonferroni-adjusted significance tests (corrected p value) and uncorrected p value by the number of comparisons made in BDNF protein and mRNA levels in the ACC
