# Supplementary material for: Regular Music Exposure in Juvenile Rats Facilitates Conditioned Fear Extinction and Reduces Anxiety after Foot Shock in Adulthood
Source: Biomed Res Int. 2019 Jul 14;2019:8740674. doi: 10.1155/2019/8740674 (PMC6662454; doi:10.1155/2019/8740674)
Supplement: Supplementary Materials — Table-S1: the percentage of time spent freezing across the 3 days of extinction training. Table-S2: the Bonferroni-adjusted significance tests (corrected p value) and uncorrected p value by the number of comparisons made in the anxiety-like behavior testing were shown as the time spent in the open arms (A), the number of open arm entries (B) in the EPM test, and the time spent in the inner area in the open field test (C). Table-S3: the Bonferroni-adjusted significance tests (corrected p value) and uncorrected p value by the number of comparisons made in BDNF protein and mRNA levels in the ACC [file 8740674.f1.pdf]

Table 1 The percentage of time spent freezing across the 3 days of extinction training (For each group, n_{male}=9, n_{female}=6). A three-way ANOVA indicated that there was a significant effect of training day and music treatment on freezing time. These data indicate that music exposure in the juvenile rats facilitated conditioned fear extinction in adulthood.

					95% Confidence Interval		Music *vs.* Control			
			**Mean**	**Std. Error**	**Lower Bound**	**Upper Bound**	**Difference**	**t**	**P value**	**95% CI of diff.**
**Day 1**	**Male**	**Music**	61.097	2.959	55.207	66.987	6.272	1.511	P > 0.05	-5.148 to 17.69
		**Control**	67.369	2.959	61.479	73.259				
	**Female**	**Music**	68.292	3.623	61.078	75.505	-17.57	3.424	P<0.01	-30.58 to -4.558
		**Control**	50.723	3.623	43.510	57.937				
**Day 2**	**Male**	**Music**	33.520	2.959	27.630	39.410	17.86	4.304	P<0.001	6.442 to 29.28
		**Control**	51.382	2.959	45.492	57.272				
	**Female**	**Music**	45.958	3.623	38.745	53.172	3.347	0.652 3	P > 0.05	-9.663 to 16.36
		**Control**	49.308	3.623	42.095	56.522				
**Day 3**	**Male**	**Music**	12.717	2.959	6.827	18.607	21.86	5.268	P<0.001	10.44 to 33.28
		**Control**	34.576	2.959	28.686	40.465				
	**Female**	**Music**	22.772	3.623	15.558	29.985	13.70	2.670	P < 0.05	0.6900 to 26.71
		**Control**	33.638	3.623	26.425	40.852				

Table 2 The Bonferroni-adjusted significance tests (corrected p-value) and uncorrected p-value by the number of comparisons made in the anxiety-like behavior testing were shown as the time spent in the open arms (A), the number of open arm entries (B) in the EPM test, and the time spent in the inner area in the open field test (C). Only pairwise comparisons of p <0.05 after ANOVA analyses present a corrected p-value. When the product of the LSD p-value and the number of comparisons exceeds 1, the Bonferroni-corrected p-value reported by SPSS will be 1.000.

	**A. After Shock**		**B. After Shock**		**C. After Shock**	
	**Uncorrected (p values)**	**Corrected (p values)**	**Uncorrected (p values)**	**Corrected (p values)**	**Uncorrected (p values)**	**Corrected (p values)**
**Male Control *vs* Female Control**	0.021	0.126	0.116	0.696	0.403	1.000
**Male Control *vs* Male Music**	0.002	0.012	0.041	0.246	0.017	0.102
**Male Control *vs* Female Music**	0.001	0.006	0.000	0.000	0.003	0.018
**Female Control *vs* Male Music**	0.000	0.000	0.001	0.006	0.004	0.024
**Female Control *vs* Female Music**	0.000	0.000	0.000	0.000	0.001	0.006
**Male Music *vs* Female Music**	0.390	1.000	0.014	0.084	0.349	1.000

Table 3 The Bonferroni-adjusted significance tests (corrected p-value) and uncorrected p-value by the number of comparisons made in BDNF protein and mRNA levels in the ACC. Only pairwise comparisons of p <0.05 after ANOVA analyses present a corrected p-value. When the product of the LSD p-value and the number of comparisons exceeds 1, the Bonferroni-corrected p-value reported by SPSS will be 1.000.

	**BDNF protein after fear conditioning**		**BDNF mRNA after fear conditioning**	
	**Uncorrected (p values)**	**Corrected (p values)**	**Uncorrected (p values)**	**Corrected (p values)**
**Male Control *vs* Female Control**	1.000	1.000	0.98	1.000
**Male Control *vs* Male Music**	0.008	0.048	0.012	0.072
**Male Control *vs* Female Music**	0.011	0.066	0.014	0.084
**Female Control *vs* Male Music**	0.011	0.066	0.011	0.066
**Female Control *vs* Female Music**	0.015	0.09	0.013	0.078
**Male Music *vs* Female Music**	1.000	1.000	0.937	1.000
